# Epidemiology of fatal and hospitalised injuries among youth in Fiji (TRIP 15)

**DOI:** 10.1111/jpc.13250

**Published:** 2016-08-27

**Authors:** Josephine Herman, Roshini Peiris‐John, Iris Wainiqolo, Berlin Kafoa, Paul Laginikoro, Eddie McCaig, Shanthi Ameratunga

**Affiliations:** ^1^ Section of Epidemiology and Biostatistics, School of Population HealthUniversity of AucklandAucklandNew Zealand; ^2^ College of Medicine, Nursing and Health SciencesFiji National UniversitySuvaFiji

**Keywords:** accident, adolescent, developing country, Pacific Island, wound and injury

## Abstract

**Aim:**

To determine the burden and characteristics of fatal and hospitalised injuries among youth in Fiji.

**Methods:**

We conducted a cross‐sectional analysis of the Fiji Injury Surveillance in Hospitals database – a prospective population‐based trauma registry – to examine the incidence and epidemiological characteristics associated with injury‐related deaths and hospital admissions among youth aged 15–24 years. The study base was Viti Levu, Fiji, during the 12‐month period concluding on 30 September 2006.

**Results:**

One in four injuries in the Fiji Injury Surveillance in Hospitals database occurred among youth (*n* = 515, incidence rate 400/100 000). Injury rates were higher among men, those aged 20–24 years compared with 15‐ to 19‐year‐olds, and indigenous Fijians (iTaukei) compared with Indians. The leading causes among indigenous Fijians were being hit by a person/object (men) and falls (women), whereas for Indians, it was road traffic injuries (men) and intentional poisoning (women). Most injuries occurred at home (39%) or on the road (22%). Of the 63 fatal events, 57% were intentional injuries, and most deaths (73%) occurred prior to hospitalisation. Homicide rates were four times higher among indigenous Fijians than Indians, whereas suicide rates were five times higher among Indians compared with indigenous Fijians.

**Conclusions:**

Important ethnic‐specific differences in the epidemiology of fatal and serious non‐fatal injuries are apparent among youth in Fiji. Efforts to prevent the avoidable burden of injury among Fiji youth thus requires inter‐sectoral cooperation that takes account of important sociocultural, environmental and health system factors such as unmet mental healthcare needs and effective pre‐hospital trauma services.

## What is already known on this topic

1. Of the approximately five million injury deaths per annum, over 90% occur in low‐ and middle‐income countries.
2. Youth aged 15 to 24 years account for 16% of injury fatalities worldwide, the commonest causes being road traffic injuries, self‐harm and violence.
3. Contemporary published literature on the overall epidemiology of injuries among youth living in the Pacific Islands is sparse.

## What this paper adds

1. Youth aged 15–24 years account for a quarter of all injuries resulting in death or hospital admission in Viti Levu, Fiji.
2. Three‐quarters (73%) of the injury deaths in this age group occurred prior to hospital presentation.
3. Injury prevention efforts must take account of sociocultural factors that may account for important differences in the epidemiology of injuries in the two major ethnic groups with indigenous Fijian (iTaukei) youth having higher rates of injury overall and assault/homicide events while Indian youth having five times higher rates of self‐harm.

Of the 5.1 million injury‐related deaths reported worldwide, 16% occur in the 15‐ to 24‐year age group.^1^ Accounting for almost half (45%) of all deaths among adolescents and youth,^2^, ^3^ injuries are gaining long‐overdue global attention as a major public health problem for this age group.^2^, ^4^, ^5^, ^6^ This is of particular importance in low‐ and middle‐income countries, which experience over 90% of injury deaths,^1^ and where youth mortality rates (particularly among men) have been static or increasing over recent decades.^7^

Despite the disproportionate burden of injuries borne by youth in low‐ and middle‐income countries, injury prevention efforts in these settings are hindered by significant gaps in knowledge regarding associated epidemiological characteristics and contextual factors.^8^, ^9^ This places, these young people and their families at risk of being trapped in poverty, with adverse societal effects in terms of escalating health‐care costs and impacts on productivity and economic development.^10^

These are particularly acute challenges for vulnerable economies in the Pacific region where youth make up one‐fifth of the total population (1.6 million) in the 22 Pacific Island countries and territories. With the majority of these Pacific nations categorised as low‐ or middle‐income and a projected rapid increase in the youth population over the next two decades, proactive measures are required to ensure their health and wellbeing.^11^, ^12^, ^13^, ^14^

Research on the burden of injuries among youth in Pacific Island countries and territories has largely focussed on descriptive studies examining self‐harm and its attendant risks.^15^, ^16^, ^17^, ^18^ This reflects broader concerns regarding suicide rates in the Pacific region, which are among the highest in the world.^19^, ^20^, ^21^, ^22^, ^23^ To the best of our knowledge, there are no published studies from a less‐resourced Pacific Island country providing an epidemiological overview of all fatal and hospitalised injuries among youth. We considered such an overview as particularly important in Fiji, the second most populous Pacific Island country, as the country's ethnic composition could result in distinct and more complex injury profiles that require specific consideration in national injury prevention efforts. Viti Levu is the main island in Fiji and home to 70% of the Fiji population. In the 2007 census, youth aged 15–24 years (*n* = 130 000) made up one‐fifth of the Viti Levu population. Indigenous Fijians (iTaukei) and Indians comprised 55% and 39% of the youth population respectively.^24^

We conducted a secondary analysis of data gathered in a prospective population‐based surveillance system to quantify the incidence‐associated epidemiological characteristics of fatal and hospitalised injuries among youth aged 15 to 24 years in Viti Levu. This analysis complements the information previously published on the epidemiology of childhood injuries in Fiji.^25^

## Methods

The Fiji Injury Surveillance in Hospitals (FISH) database was a study‐specific prospective trauma registry established in all trauma‐admitting hospitals in Viti Levu, Fiji, as part of the traffic‐related injury in the Pacific (TRIP) research project. Using a methodology previously described^26^ and a pre‐defined set of variables recommended by the World Health Organization injury surveillance guidelines,^27^ the FISH database recorded information on the demographic and associated characteristics of injury deaths and hospital admissions for 12 h or more, over a 12‐month period up to 30 September 2006. Eligible cases were identified from hospital accident and emergency registers, admission records and mortuary registers. Data recorded included: the reported intent and mechanism of injury, nature of the principal injury, activity and location of injury, circumstances (conflict situation, leisure/play/sport, travel, work, other) and the clinical outcome (fatal, non‐fatal). The variable ‘circumstances’ related to the particular activity the injured person was engaged in or exposed to at the time of the injury. A conflict situation was defined as a disagreement (verbal or physical) with another person and/or a situation consistent with a significant psychological stressor.

We also sought data on the use of alcohol, kava (a root plant with anxiolytic and sedative properties commonly consumed in Fiji)^28^, ^29^ and other substances, in the 6 h prior to the injury. Cases eligible for analysis in this study were all injury presentations in the FISH database of youth aged 15 to 24 years.

We analysed data using Microsoft^®^ Excel Version 2010, and STATA^®^ Version 12 statistical software. Population data from the Fiji census (2007)^24^ was used to estimate injury incidence rates. This study was approved by the Fiji National Research Ethics Review Committee of the Ministry of Health.

## Results

The 515 injury events among youth aged 15–24 years accounted for 24% of all injuries recorded in the FISH database, and an annual injury incidence rate for youth of 400/100 000 population. The major causes of injury were being hit by a person or object (27%), falls (18%), road traffic injury (RTI) (17%) and poisoning (13%). Intentional injuries (self‐harm or interpersonal violence) accounted for 36% of injuries. Common types of injury were fractures (32%), cuts/open wounds (22%) and head injuries (11%). Most injuries occurred in homes (39%), highways/roads (22%) and recreational areas (14%). Although 14% and 3% of youth reported consuming alcohol and kava, respectively within 6 h of the injury, the high rate of missing information (20% and 26% respectively) made it difficult to interpret these results.

### Injury profiles by age group and ethnicity

**Figure 1:**
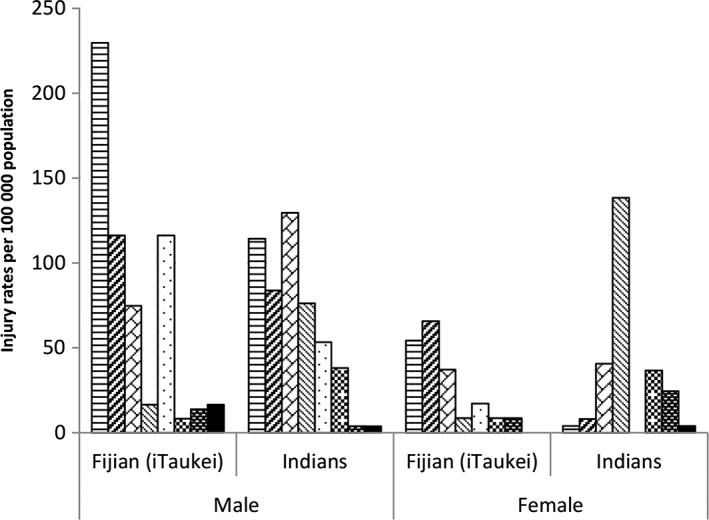
Fiji youth injury rates per 100 000 population, by cause, sex and ethnic group. (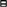), Hit by person or object; (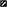), fall; (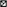), road trafic injury; (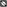), poisoning; (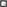), stab/cut; (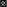), choking/hanging; (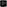), fire/heat/electricity; (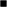), drowning.

In general, injury rates were higher among older youth (20 to 24 years) compared with those aged 15 to 19 years and twice as high among men compared with women (Table 1). While overall injury rates were higher among indigenous Fijians compared with Indians, there were important ethnic‐specific differences. Injuries arising from ‘leisure, play, or sporting activities’ were more common among indigenous Fijians, compared with a ‘conflict situation’ among Indians. The rate of self‐harm was five times higher among Indians while interpersonal violence‐related injury was four times higher among indigenous Fijians. The leading causes of injury for indigenous Fijian men were being hit by a person or object, falls and stabs/cuts. Among Indian men, these were RTI, being hit by a person or object and falls (Fig. 1). Poisonings were the commonest cause among Indian women, while falls and being hit by a person/object were leading causes among indigenous Fijian women.

**Table 1:** Incidence rates (per 100 000) of fatal and hospitalised injuries by age and ethnicity (1 October 2005–30 September 2006)

Demographic and injury characteristics		Age group						Ethnic group					
		15–19 years (*n* = 196)			20–24 years (*n* = 319)			Fijian (iTaukei) (*n* = 293)			Indian (*n* = 197)		
		*n*	Rate	(95% CI)	*n*	Rate	(95% CI)	*n*	Rate	(95% CI)	*n*	Rate	(95% CI)
Total		196	311.9	(268.3–355.6)	319	482.1	(429.2–535.0)	293	411.9	(364.7–459.1)	197	387.7	(333.5–441.8)
Sex	Male	140	434.2	(362.3–506.2)	230	680.6	(592.6–768.5)	219	606.0	(525.8–686.3)	133	506.6	(420.5–592.7)
	Female	56	183.1	(135.1–231.0)	89	274.9	(217.8–332.0)	74	211.5	(163.3–259.6)	64	260.6	(196.7–324.4)
Age (years)	15–19							120	334.0	(274.2–393.8)	67	285.0	(216.7–353.2)
	20–24							173	491.4	(418.2–564.7)	130	476.1	(394.3–557.9)
Intent	Unintentional	128	203.7	(168.4–239.0)	183	276.6	(236.5–316.6)	192	269.9	(231.7–308.1)	104	204.7	(165.3–244.0)
	Intentional	64	101.9	(76.9126.8)	120	181.4	(148.9–213.8)	90	126.5	(100.4–152.7)	85	167.3	(131.7–202.8)
	Interpersonal violence	23	36.6	(21.6–51.6)	66	99.7	(75.7–123.8)	71	99.8	(76.6–123.0)	14	27.6	(13.1–42.0)
	Self‐harm	41	65.3	(45.3–85.2)	54	81.6	(59.8–103.4)	19	26.7	(14.7–38.7)	71	139.7	(107.2–172.2)
Cause of injury	Hit by person or object	43	68.4	(48.0–88.9)	95	143.6	(114.7–172.4)	102	143.4	(115.6–171.2)	31	61.0	(39.5–82.5)
	Fall	49	78.0	(56.2–99.8)	48	72.5	(52.0–93.1)	65	91.4	(69.2–113.6)	24	47.2	(28.3–66.1)
	Road traffic injury	25	39.8	(24.2–55.4)	62	93.7	(70.4–117.0)	40	56.2	(38.8–73.7)	44	86.6	(61.0–112.2)
	Poisoning	28	44.6	(28.1–61.1)	38	57.4	(39.2–75.7)	9	12.7	(4.4–20.9)	54	106.3	(77.9–134.6)
	Stab/cut	21	33.4	(19.1–47.7)	42	63.5	(44.3–82.7)	48	67.5	(48.4–86.6)	14	27.6	(13.1–42.0)
	Choking/hanging	11	17.5	(7.2–27.9)	16	24.2	(12.3–36.0)	6	8.4	(1.7–15.2)	19	37.4	(20.6–54.2)
	Drowning	7	11.1	(2.9–19.4)	3	4.5	(0.0–9.7)	6	8.4	(1.7–15.2)	2	3.9	(0.0–9.4)
Nature of injury	Fracture	62	98.7	(74.1–123.2)	102	154.2	(124.2–184.1)	97	136.4	(109.2–163.5)	56	110.2	(81.3–139.1)
	Cut/bite, open wound	39	62.1	(42.6–81.6)	76	114.9	(89.0–140.7)	90	126.5	(100.4–152.7)	22	43.3	(25.2–61.4)
	Head injury/concussion	17	27.1	(14.2–39.9)	41	62.0	(43.0–80.9)	37	52.0	(35.3–68.8)	21	41.3	(23.7–59.0)
	Asphyxia	17	27.1	(14.2–39.9)	17	25.7	(13.5–37.9)	12	16.9	(7.3–26.4)	19	37.4	(20.6–54.2)
	Sprain/strain/dislocation/bruise	18	28.6	(15.4–41.9)	24	36.3	(21.8–50.8)	28	39.4	(24.8–53.9)	12	23.6	(10.3–37.0)
Circumstance	Conflict situation	45	71.6	(50.7–92.5)	99	149.6	(120.1–179.1)	66	92.8	(70.4–115.2)	70	137.8	(105.5–170.0)
	Leisure/play/sport	85	135.3	(106.5–164.0)	72	108.8	(83.7–133.9)	111	156.1	(127.0–185.1)	38	74.8	(51.0–98.6)
	Travel	27	43.0	(26.8–59.2)	69	104.3	(79.7–128.9)	52	73.1	(53.2–93.0)	41	80.7	(56.0–105.4)
	Work	27	43.0	(26.8–59.2)	51	77.1	(55.9–98.2)	49	68.9	(49.6–88.2)	26	51.2	(31.5–70.8)
	Other/unknown	12	19.1	(8.3–29.9)	28	42.3	(26.6–58.0)	15	21.1	(10.4–31.8)	22	43.3	(25.2–61.4)

### Fatal injuries

Twelve per cent (*n* = 63) of injury events were fatal, and almost three‐quarters (*n* = 46) of these deaths occurred before hospital admission. Among indigenous Fijians, fatal injuries were more common among men while no gender difference was apparent among Indians (Table 2). Two‐thirds of all fatalities occurred in the 20‐ to 24‐year age group, most of whom were Indians. The majority of fatalities were intentional (57%) with a high proportion among Indian youth (70%), largely because of self‐harm. While choking/hanging and RTI were the leading causes of injury deaths among indigenous Fijian and Indian youth, drowning was also an important cause among indigenous Fijians.

**Table 2:** Frequency of fatal and non‐fatal hospitalised injuries among Fiji youth, by ethnic group (1 October 2005–30 September 2006)

		Non‐fatal hospitalised injuries (*n* = 452)			Fatal injuries (*n* = 63)		
		Total (*n* = 452)	Fijian (iTaukei) (*n* = 272)	Indian (*n* = 160)	Total (*n* = 63)	Fijian (iTaukei) (*n* = 21)	Indian (*n* = 37)
Sex	Male	334 (73.9)	205 (75.4)	114 (71.3)	36 (57.1)	14 (66.7)	19 (51.4)
	Female	118 (26.1)	67 (24.6)	46 (28.8)	27 (42.9)	7 (33.3)	18 (48.7)
Age (years)	15–19	174 (38.5)	109 (40.1)	58 (36.2)	22 (34.9)	11 (52.4)	9 (24.3)
	20–24	278 (61.5)	163 (59.9)	102 (63.8)	41 (65.1)	10 (47.6)	28 (75.7)
Intent	Unintentional	286 (63.3)	179 (65.8)	94 (58.8)	25 (39.7)	13 (61.9)	10 (27.0)
	Intentional	148 (32.7)	83 (30.5)	59 (36.9)	36 (57.1)	7 (33.3)	26 (70.3)
	Interpersonal violence	88 (19.5)	70 (25.7)	14 (8.8)	1 (1.6)	1 (4.8)	0
	Self‐harm	60 (13.3)	13 (4.4)	45 (28.1)	35 (55.6)	6 (28.6)	26 (70.3)
	Undetermined/unknown	18 (4.0)	10 (3.4)	7 (4.4)	2 (3.2)	1 (4.8)	1 (2.7)
Cause	Hit by person or object	137 (30.3)	101 (37.1)	31 (19.4)	1 (1.6)	1 (4.8)	0
	Fall	95 (21.0)	63 (23.2)	24 (15.0)	2 (3.2)	2 (9.5)	0
	Road traffic injury	74 (16.4)	35 (12.9)	36 (22.5)	13 (20.6)	5 (23.8)	8 (21.6)
	Poisoning	60 (13.3)	9 (3.3)	50 (31.3)	6 (9.5)	0	4 (10.8)
	Stab/cut	62 (13.7)	48 (17.7)	13 (8.1)	1 (1.6)	0	1 (2.7)
	Choking/hanging	2 (0.4)	0	1 (0.6)	25 (39.7)	6 (28.6)	18 (48.7)
	Fire/heat/electrical	10 (2.2)	7 (2.6)	3 (1.9)	5 (7.9)	1 (4.8)	4 (10.8)
	Drowning	0	0	0	10 (15.9)	6 (28.6)	2 (5.4)
	Sexual assault, other, unknown	12 (2.7)	9 (3.3)	2 (1.3)	0	0	0
Circumstance	Conflict situation	117 (25.9)	61 (22.4)	51 (31.9)	27 (42.9)	5 (23.8)	19 (51.4)
	Leisure/play/sport	147 (32.5)	105 (38.6)	36 (22.5)	10 (15.9)	6 (28.6)	2 (5.4)
	Travel	83 (18.4)	46 (16.9)	34 (21.3)	13 (20.6)	6 (28.6)	6 (18.9)
	Work	75 (16.6)	46 (16.9)	26 (16.3)	3 (4.8)	3 (14.3)	0
	Other/unknown	30 (6.6)	14 (5.2)	13 (8.1)	10 (15.9)	1 (4.8)	9 (24.3)
Location	Private house/compound	162 (35.8)	80 (29.4)	73 (45.6)	36 (57.1)	8 (38.1)	25 (67.6)
	Highway/road	98 (21.7)	58 (21.3)	36 (22.5)	14 (22.2)	6 (28.6)	8 (21.6)
	Area of recreation	65 (14.4)	52 (19.1)	11 (6.9)	5 (7.9)	2 (9.5)	1 (2.7)
	School	18 (4.0)	14 (5.2)	4 (2.5)	1 (1.6)	0	1 (2.7)
	Work place	52 (11.5)	31 (11.4)	19 (11.9)	1 (1.6)	1 (4.8)	0
	Other	17 (3.8	12 (4.4)	5 (3.1)	4 (6.4)	3 (14.3)	1 (2.7)
	Unknown	40 (8.9)	25 (9.2)	12 (7.5)	2 (3.2)	1 (4.8)	1 (2.7)
Nature	Fracture	161 (35.6)	94 (34.6)	56 (35.0)	3 (4.8)	3 (14.3)	0
	Cut/bite, open wound	114 (25.2)	90 (33.1)	21 (13.1)	1 (1.6)	0	1 (2.7)
	Head injury/concussion	48 (10.6)	33 (12.1)	15 (9.4)	10 (15.9)	4 (19.1)	6 (16.2)
	Asphyxia	0	0	0	34 (54.0)	12 (57.1)	19 (51.4)
	Sprain/strain/dislocation/bruise	42 (9.3)	28 (10.3)	12 (7.5)	0	0	0
	Burn	7 (1.6)	4 (1.5)	3 (1.9)	5 (7.9)	1 (4.8)	4 (10.8)
	Internal injuries chest/abdomen	8 (1.8)	4 (1.5)	3 (1.9)	3 (4.8)	0	2 (5.4)
	Other/unknown	72 (15.9)	19 (7.0)	50 (31.3)	7 (11.1)	1 (4.8)	5 (13.5)

### Non‐fatal hospital admissions

One‐third of non‐fatal injuries were deemed intentional with interpersonal violence being more common among indigenous Fijians and self‐harm more common among Indians (Table 2). Indigenous Fijian youth were more commonly injured after being hit by a person/object or falling, sustained most injuries at home, on the road and recreational areas, with the circumstances of injury most commonly attributed to leisure, play and sporting activities. In contrast, Indian youth were more commonly injured from poisoning and RTI.

## Discussion

In this population‐based study from Fiji, youth aged 15 to 24 years accounted for one in four serious injuries resulting in death or hospital admission for 12 h or more. In general, injury rates were higher among men and older youth. Indigenous Fijians had higher overall rates of injury (i.e. deaths and hospital admissions combined) but Indians had higher rates of fatal injury. Among indigenous Fijians, injured men were most commonly hit by a person/object while women were injured in falls. Among Indians, men were most often injured in road crashes while (intentional) poisoning comprised the commonest injury among women. Most fatal injury events occurred at home, and three‐quarters of injury deaths occurred prior to hospital admission. Almost two‐thirds of fatal injuries were intentional, the majority of which were due to self‐harm among Indians. Rates of youth suicide among Indians were five times that of indigenous Fijians.

The prospective trauma registry established as part of the wider TRIP project in Viti Levu, Fiji, provided a unique opportunity to investigate the population‐based epidemiology of life‐threatening injuries among youth. By including data from mortuaries as well as all trauma‐admitting hospitals, the study also addressed some common biases in hospital or trauma centre‐based studies, which do not typically capture data on pre‐hospital deaths and deny the opportunity to estimate population‐based incidence of injury. Applying this approach in Fiji helped distinguish ethnic‐specific differences in the epidemiology of serious injuries among Fiji youth.

Notwithstanding these strengths, our findings must be interpreted in light of several limitations. The precision of the estimates is limited by the relatively small size of the population base, and the limited period of FISH data collection (12 months). The latter also precluded the ability to examine year to year variations and trends over time. While a standardised quality‐assured data collection method was employed using World Health Organization guidelines for less‐resourced settings,^26^, ^30^ the data accessible from clinical records precluded an analysis of details such as the types of road users involved in RTI or degrees of exposure to environmental hazards. Furthermore, while we generated a broad profile of injuries resulting in death or hospital admissions for 12 h or more (most serious injuries are considered likely to present to hospitals in Fiji), less severe injuries (in terms of immediate threat to life) were outside the scope of this study. Injuries that are not life threatening are more common in the community and can result in disabling consequences, substantial out‐of‐pocket costs and attendant stresses to young people and their families.

The high rate of interpersonal violence among indigenous Fijians in this study is similar to findings from a population‐based self‐report survey of students aged 14 to 17 years in three Pacific Islands (Pohnpei, Tonga, Vanuatu) in which 24 to 62% of participants reported experiencing intentional physical injuries (by implication, these referred to interpersonal violence) in the preceding 12 months.^31^ Factors associated with these injuries included school bullying and the use of tobacco (Tonga, Vanuatu) and illicit drugs (Pohnpei, Vanuatu).

The high rates of fatalities by choking or hanging among Indians in this study is consistent with previous studies focussing on suicide in Fiji.^17^, ^32^ In a comparative study of suicides in Pacific Island countries and territories, the highest rates were observed in youth from Fiji of Indian ethnicity, the Chuuk State of the Federated States of Micronesia, Samoa and Guam, with overall rates higher among men, except among Fiji‐Indian and Samoan women.^32^ The preponderance of injuries occurring in the home environment also reflects a high proportion of self‐harm events in this setting, a finding consistent with a study examining Pacific youth suicide in Auckland, New Zealand.^33^

Our study was not designed to explore the specific circumstances surrounding injuries, such as those occurring in the context of sport and recreation, violence or self‐harm among Fiji youth. However, our findings alongside the existing literature reveal the need to explore the complex sociocultural, geopolitical, intergenerational and environmental factors that require attention to address this avoidable mortality.^22^, ^31^, ^32^, ^34^, ^35^ It has been reported that a high proportion of children in Fiji experience direct physical punishment in Fiji,^36^ a factor that could be associated with injuries relating to interpersonal violence.^35^ Other researchers attribute the high rates of suicides among young Indians to intergenerational and family conflicts relating to peer relationships and marriage.^17^, ^37^ While the specific sociocultural contexts and other associated characteristics may vary among both indigenous Fijian and Indian communities, the roles that parents, families and caring adults could play in interrupting cycles of inter‐ and intrapersonal violence require greater attention. Furthermore, the availability and access to culturally appropriate, youth‐friendly, mental health and counselling services should be a high priority. These are aspects of health care which have historically been poorly resourced in the Pacific Islands.^17^, ^19^, ^22^ Given most injury deaths (74%) occurred prior to hospital admission, access to high quality pre‐hospital care and emergency response services also requires urgent attention.

Findings regarding RTIs are generally consistent with studies from Papua New Guinea and the Pacific region in terms of the high proportion of young male crash victims and pre‐hospital deaths.^38^ The relatively low number of workplace injuries while possibly reflecting a lower youth employment rate in these settings requires further investigation. Similarly, for indigenous Fijians, serious injuries in the context of sport and recreation would benefit from context‐appropriate evidence‐based preventative measures.

Previous studies of RTIs and self‐harm in the Pacific suggest alcohol to be an important contributor, especially among injuries involving young men.^22^, ^38^, ^39^ Harm reduction approaches are likely to be challenged by the sale, supply and availability of alcohol in poorly regulated environments in the Pacific Islands. Some international regulatory authorities have cautioned against consuming kava or products containing kava and driving or using heavy machinery, but the associations involved require more rigorous evaluation, particularly in contexts where recreational consumption of kava is common.^40^

## Conclusion

Despite the disproportionate burden of injury borne by youth in low‐ and middle‐income countries, there are remarkable gaps in infrastructure and service investment in youth health, social, educational and economic policies in these settings.^8^, ^9^ The complex sociopolitical issues confronting small Pacific Island nations compound the perils of neglect for youth in these settings. National and regional efforts should focus on culturally appropriate injury control interventions that take account of underlying determinants and stresses experienced by vulnerable youth in Pacific Islands, while also drawing on their sources of strength and resilience.^6^, ^17^, ^32^, ^41^ Periodic community surveys of key risk and protective factors for injury could usefully inform context‐specific prevention initiatives.^42^, ^43^, ^44^ Effective injury and risk factor surveillance systems can also serve as vital monitoring instruments to evaluate the impact of interventions designed to reduce premature death, morbidity and disability among Pacific youth.^10^, ^18^ This study highlights the need for intersectoral efforts that achieve these objectives as a regional health priority.

Although the data were collected a decade ago, our study has added critical injury information not previously available to Fiji and the Pacific region. To our knowledge, there have been no major changes in the epidemiology of injuries because the collection of these data and the rates of fatal injury events for this age group captured by the Fiji Ministry of Health have remained relatively constant. Consequently, this study continues to inform current Pacific Island country road safety initiatives including the Cook Islands Road Safety Strategy 2016–2020 and the World Health Organization Regional Action Plan for Violence and Injury Prevention in the Western Pacific (2016–2020).
